# Acoustically Controlled Binaural Auditory Training with Vocal Duets: Assessment and Effectiveness

**DOI:** 10.6061/clinics/2021/e2085

**Published:** 2021-03-15

**Authors:** Taís de Azevedo Picinini, Simone Sperança, Liliane Desgualdo Pereira

**Affiliations:** I Programa de Pos-Graduacao em Disturbios da Comunicacao Humana, Universidade Federal de Sao Paulo, Sao Paulo, SP, BR.; II Departamento de Fonoaudiologia, Escola Paulista de Medicina, Universidade Federal de Sao Paulo, Sao Paulo, SP, BR.

**Keywords:** Auditory Perception, Hearing Tests, Auditory Perceptual Disorders, Neuronal Plasticity, Rehabilitation, Vocal Melody

## Abstract

**OBJECTIVES:**

We aimed to evaluate the effectiveness of a binaural auditory training program with vocal duets by comparing skills through outcomes from behavioral and electrophysiological assessment instruments at three moments: before the intervention, moment one (M1); immediately after training, moment two (M2); and 3 months after, moment three (M3).

**METHODS:**

This interventional, longitudinal, prospective, and uncontrolled study was approved by our Research Ethics Committee. Binaural auditory training with vocal duets (ATVD) was applied in 10 adults with normal audiometric thresholds and auditory processing disorders. ATVD used four different vocals of a public domain song sung in *a* *cappella* as stimuli. Participants were asked to register any perceived difference in frequency for each syllable of the song during 30-minute sessions twice a week. The number of sessions required ranged from 12 (6 hours) to 20 (10 hours).

**RESULTS:**

Regarding behavioral tests, the dichotic consonant-vowel test showed significant evidence of an improved advantage in the left ear (LE) in the non-forced condition and a significant reduction in the number of errors at M2 and M3 in the forced left condition. The speech-in-noise test and frequency pattern test showed a significant reduction in impaired results at M2 and M3. Electrophysiological results showed a significant increase in the LE amplitude in the P3 long-latency auditory evoked potentials test, as well as a decrease in the auditory brainstem response test (III-V and I-V inter-peak latencies in the right ear and wave I and I-III inter-peak latencies in LE).

**CONCLUSION:**

The effectiveness of ATVD was evidenced, and the results were maintained after 3 months.

## INTRODUCTION

Binaural processing and temporal processing are elementary mechanisms of a complex set of cognitive and neurophysiological processes called central auditory processing (CAP) (1). Binaural processing allows people to understand speech in everyday and noisy listening environments and perform sound localization (2,3). On the other hand, temporal processing is the basis of auditory processing since several characteristics of auditory information are influenced by time (4).

In this sense, the musical experience is an effective resource for the stimulation of binaural processing and improvement of temporal processing skills, as it stimulates the development of auditory perception, melody, and harmony through perceptual training of intervals, rhythm, and other acoustic parameters (5). In music, intervals are the distances between the notes on a given musical scale. They can be melodic (one note after another) or harmonic (when the two notes are sung or played at the same time) as it happens, for example, in a vocal duet (6).

In a vocal duet, when one individual sings on a certain note, the other voice sings notes above or below the dominant voice, and the melodic lines of each of these voices are arranged at intervals that are harmonious (7). Such harmonization between the melodies requires a refinement of the singers’ auditory skills. In addition, singers show increased activation of their bilateral primary somatosensory cortex, lower parietal lobe, and dorsolateral prefrontal cortex, and increased activation in the basal ganglia at the subcortical level, thalamus, and cerebellum, which corroborates the idea that brain plasticity is influenced by musical training (8).

Given the need for more tools in clinical practice to develop binaural auditory and temporal processing, the use of musical stimuli is an effective resource in the reorganization of brain connections (5). Therefore, this study aimed to evaluate the effectiveness of a binaural auditory training program with vocal duets based on the characterization and comparison of skills before and after the therapeutic intervention using outcomes from behavioral and electrophysiological assessment instruments.

## METHODS

The study was approved by our institutional ethics committee under protocol number 2,188,930 and was carried out in the neuroaudiology service. The sample used in this study comprised 10 university students (19 to 33 years old; 70% female) to ensure that they had the cognitive and abstraction level required to perform the tasks. The complaints reported by the individuals were inattention, difficulty in understanding noise, difficulty in learning other languages, and/or engaging in musical activities.

### Inclusion criteria

The volunteers of the sample were all right-handed and 18 years and over. They referred to diverse communication difficulties and presented hearing thresholds of up to 25 dBHL at frequencies from 250 to 8000 Hz, with a type A or normal tympanogram test; absence of evidence or self-report of neurological and/or cognitive impairments; and no previous musical experience, according to information collected from their medical history. The participants who passed the inclusion criteria completed behavioral and electrophysiological tests to evaluate CAP. To diagnose central auditory processing disorder (CAPD), they had to present at least two impaired tests. Moreover, all participants had to agree to attend the therapeutic intervention sessions.

### Assessment instruments

The auditory processing assessment was carried out through behavioral and electrophysiological tests in the following three moments: before the intervention, moment 1 (M1); immediately after the intervention, moment two (M2); and 3 months after the intervention, moment three (M3). The time for each assessment was approximately 2 hours.

The behavior tests were performed using an audiometer in a soundproof booth. Four behavioral tests and abilities were used to evaluate CAP.

- Temporal ordering skill of brief and successive sounds: frequency pattern test (FPT). Thus test was applied in a binaural form, and the normality criterion for this sample was ≥76% accuracy (10).
- Temporal resolution skill: random gap detection test (RGDT). This test was applied in a binaural form. The normality criterion for this sample was ≤10 ms (11).
- Closing skill: speech weighted noise (SWN). This test was applied in a monotic form using a speech-in-noise test (SNT). The normality criterion was a percentage of correct responses in the SNT ≥70% and the difference between SNT and Word Discrimination Test (WDT) up to <20%, equivalent to a normal SWN (12).
- Figure-ground auditory skill for verbal sounds: Consonant-vowel signals in dichotic listening (DL). The dichotic consonant-vowel test (DCVT) with three different condition tasks was used. In this, the participant was directed to report which syllable was most clearly heard (non-forced, NF) and which syllable was presented to each ear, the right [forced right (FR)] and left [forced left (FL)]. The normality criterion for the sample was 19 correct syllables and advantage the right ear, in the NF condition. In the FR condition, it was two or more correct syllables in the right ear regarding the NF condition and a maximum of five errors. In the FL condition, it was four or more correct syllables regarding the NF condition and a maximum of five errors (12).

Electrophysiological evaluation was performed on a Smart EP, Intelligent Hearing Systems^®^ device, according to an electrophysiological evaluation protocol (13) and was performed in a silent room in partial darkness. Participants were instructed to remain still and relaxed throughout the three tests. The long-latency auditory evoked potentials (LLAEP), frequency following response (FFR), and auditory brainstem response (ABR), were carried out in the following order as explained below:

- The LLAEP was obtained using an oddball paradigm with tone burst stimulus. Frequent and rare stimuli were at 1000 Hz and 2000 Hz tones with 85% and 15% probability, respectively, and performed at 75 dBHL. The subtraction of rare from frequent tracings created a waveform from which N2 latency and P3 latency and amplitude were determined and considered. For the analysis of this potential, the normal values used were those proposed by McPherson (1996) (13).
- The FFR was obtained through two 3000 stimulus syllable /da/, with a duration of 40 ms, at 80 dBHL presented monaurally to the right ear. The resulting tracing was used to mark V, A, C, D, E, F, and O components and analyze their latency and amplitude values. In addition, the amplitude V-A complex and its slope were analyzed. For the analysis of this potential, the normal values used were those proposed by Skoe et al. (14).
- The ABR was obtained with a click stimulus presented monaurally at 80 dBHL. The absolute latencies of waves I, III, and V and the inter-peak intervals I-III, III-V, and I-V were marked and recorded after 2,000 stimuli and presented twice to analyze the reproducibility of the tracing. For the analysis of this potential, the normal values used were those from the biological calibration of the service where the research was conducted. The absolute latencies of waves I, III, and V and the inter-peak intervals I-III, III-V, and I-V are shown below in ms and their standard deviation, respectively: 1.65 ms (±0.06); 3.80 ms (±0.15); 5.67 ms (±0.16); 2.15 ms (±0.16); 1.86 ms (±0.12); 4.01 ms (±0.17).

### Therapeutic Intervention Material - Auditory training

The tool developed for this research was designed with voices using a publicly available song entitled Peixe Vivo sung *a cappella*. The song was recorded in a studio in four voices: the first voice in the main melody was the tonic voice, (V1); the second voice, at an interval of a sixth below the tonic voice, was the low-pitched voice (V2); the third voice, at an interval of a third upward from the main voice, was the high-pitched voice (V3), and the fourth voice was one that was more monotone compared to the previous ones (V4).

Each voice was recorded on an independent channel, thereby allowing for the individual control of the stimulus level on the audiometer. Voices were presented at levels ranging from +20 dB to -20 dB to make the task more challenging as performance improved during training.

The song was divided into syllables, totaling 83 syllables. The participants were asked to draw a graphic symbol (x or 0) in the specific field on the record sheet to show their perception of changes in frequency. If the informants perceived the next syllable to have a higher pitch than the previous one, they were expected to mark the ascending symbol. On the other hand, if they perceived the next syllable to have a lower pitch compared to the previous one, they were expected to mark the descending graphic symbol, as shown in Figure 2.

**Figure 1:**
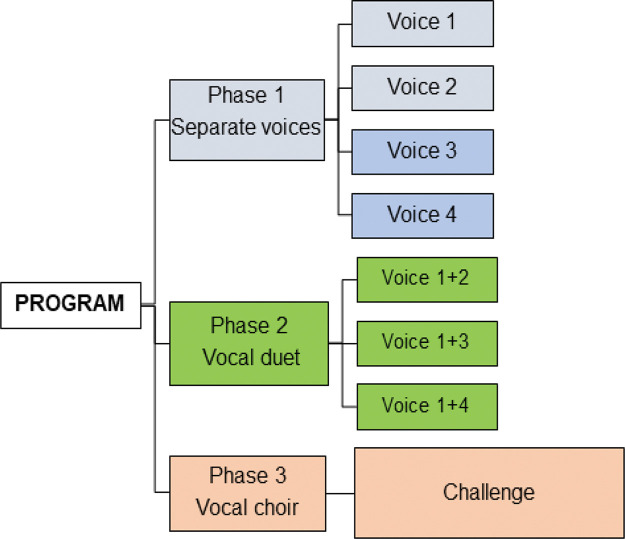
Flowchart showing the use of the auditory training program with vocal duets.

**Figure 2:**
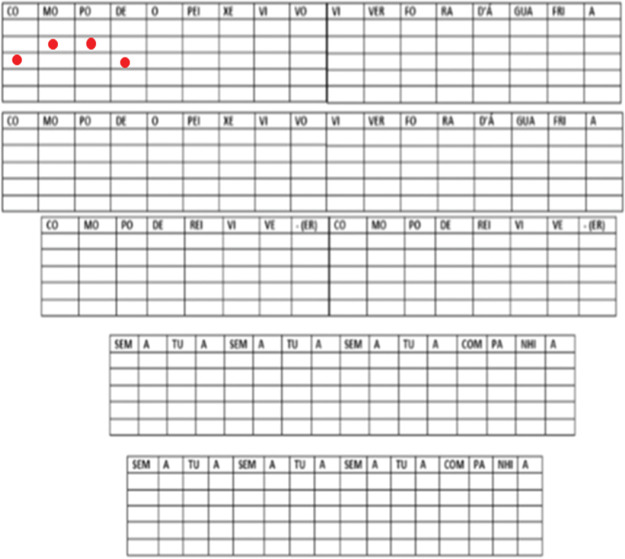
Example of marking the frequency variation perceived in the first four syllables of the song.

Before the beginning of the intervention, each participant was instructed on how to perform the task through examples given by the researcher regarding the variability of the frequency of the syllables of the music, with brief training. Afterward, they received the sheet to fill in their answers, as well as a pencil and eraser. Then, training was started inside an acoustic booth using headphones. The music was paused as required to ensure that each participant had time to mark their perception of the pitch on the answer sheet. In addition, when necessary, if a participant was unsure about a certain part of the music, then that section would be repeated as many times as necessary during the session. In all phases of the intervention, only one musical stimulus was used, that is, the same song sung in four different voices in *a cappella.* In phase one, the voices were presented separately. In phase two, they were presented in a duet, with the tonic voice always being the reference stimulus. In phase three, they were presented in chorus (all together, simultaneously) during the intervention program, as shown in Figure 1. The stimuli were always presented binaurally with dichotic stimulation. The individual did not perform any additional activities at home to avoid interferance in the assessment of the effectiveness of the auditory training with vocal duets (ATVD).

The difficulty of the task increased according to the success of the participant’s performance. In other words, the difficulty of the task depended on the number of correct answers of the informant, and it was determined according to the proposal to maintain a success rate-approximate error ratio of 70/30% (9).

The program intervention was considered complete when the last level of phase two of the ATVD was concluded. All individuals completed phase one and the last level of phase two. Phase three was presented as a bonus activity (challenge), although half of the individuals managed to respond to the most difficult rate (-20 dB) between the voices in this phase. Most participants needed a minimum of 12 acoustically controlled auditory training sessions of 30 minutes each twice a week to reach phase two of the ATVD. Those who did not reach that level after 12 sessions continued the therapy, and the number of sessions required for each participant to get to level two was recorded. After 3 months, two individuals were unable to attend the sessions. Therefore, the sample was composed of eight individuals at M3.

### Statistical Methods

Following data collection, an Excel^®^ spreadsheet was produced. To compare the performance of each ear and the difference between them, the Student’s t-test for paired samples (symmetrical distribution of quantitative variables), Wilcoxon (asymmetric distribution of quantitative variables), and McNemar (categorical variables) tests were used. The test results were compared over time using generalized estimating equations, with fit by the least significant difference test. The linear model was used for the numerical variables, and the binary logistic model was applied for categorical variables.

The level of significance was set at 5% (*p*<0.05), and the analyses were performed using the Statistical Package for the Social Sciences version 21.0.

## RESULTS

This sample comprised male and female individuals (70% female) between the ages of 19 and 33 years (mean age: 23.4±3.8 years). The number of sessions required to complete the program ranged from 12 (6 hours) to 20 (10 hours).

The test values, considering the mean and standard error has been presented in Tables 1 (DCVT) and 2 (SNT, FPT, and RGDT) for the three intervention moments: M1, M2, and M3. Table 1 shows significant evidence of the improved advantage in the left ear (LE) for the NF condition of DCVT, with performance maintained at M3. There was a significant reduction in the number of errors at M2 and M3 in the FL condition. Table 2 presents statistically significant evidence of improved performance in SNT (RE and LE) and FPT (Humming and Naming), with a reduction in the number of impaired results.

Tables 3, 4, and 5 present the means and standard errors of the parameters measured at each moment for each electrophysiological procedure: LLAEP - N2 and P3 ABR and FFR, respectively. The following results are noteworthy: a statistically significant increase in LE amplitude in P3 (Table 3), a decrease in III-V inter-peak latency and I-V inter-peak latency in RE, and in absolute latency wave I and I-III inter-peak latency in LE (Table 4). The FFR showed an increase in wave E latency from M1 to M2 and a decrease in wave E from M2 to M3 (no difference between M1 and M3). M1 had a lower mean compared to M2 and M3 (Table 5).

## DISCUSSION

Stimuli using melodic lines sung *a cappella* result in linguistic as well as musical information being combined in the same acoustic signal. This kind of stimulus supports the connections between the neural networks of the middle and superior temporal gyri and the inferior and middle frontal gyri of both hemispheres (15). Moreover, the ATVD focuses on binaural stimulation (because of the possibility of activation of the left and right hemispheres), frequency discrimination, figure-ground discrimination, sound sequencing, gap detection, and auditory attention. These are all essential components of auditory processing, as they establish the basis for more complex auditory processes (15).

In Brazil, to date, no strategies have been found to use stimuli sung in a duet for binaural stimulation to develop auditory skills in speech-language therapy. In the reviewed literature, a single international study was found that used stimuli sung *a cappella* (15). These stimuli were not used for therapeutic purposes but rather to investigate the perception of music and speech in singers and professional actors to separate and investigate the cortical networks involved in music and speech processing.

The division of songs into syllables, to facilitate the count of correct and incorrect answers and patients’ responses, could be used because Brazilian Portuguese is a syllable-timed language (16). The pitch of the notes of the melodies of all the voices of the song used in this tool ranged from 197 Hz to 332 Hz, which correspond, respectively, to the G (G3) and E (E4) notes. Thus, to make the training sessions more attractive, new songs should be created, with special attention to the pitches being used. Furthermore, various applications or software could be developed so that participants could be provided with immediate feedback on their responses. We also recommend starting phase two with more difficult signal-to-noise ratios and make phase three more challenging.

The ATVD works on subtleties in the perception of pitch. Therefore, if an individual has significant temporal processing difficulties, prior training at a less challenging level is recommended. It is also crucial to pay attention to patients’ audiogram characteristics because some auditory thresholds may be within the normal range. However, attention and discrimination of differences between low, medium, and high pitches in the same ear and between ears can interfere with binaural processing (17).

The auditory skills predominantly stimulated with the tasks in the program were temporal ordering and temporal ordering of frequencies with figure-ground skills in DL.

As for the results obtained in the behavioral tests (Tables 1 and 2), there was an improvement in the auditory skills of temporal ordering, temporal resolution, and figure-ground and an improvement in the advantage of the LE in DCVT. These findings are justified considering the relevance of binaural processing (18) in FPT and DCVT.

The bottom-up strategy improved inter-hemispheric communication (19), facilitating access to analyses of longer time frames and contributing to increased speech recognition. This is because left hemisphere activation is specialized in analyzing short speech times, such as formant processing and phoneme transition, while the right hemisphere analyzes longer time frames, such as processing the speech envelope of intonation and prosody (19).

Bottom-up processing was prevalent in ATVD, so an improvement was not expected in the directed attention tasks (FR and FL). Therefore, the reduction in the number of errors in the FL task, without changes in perceptual asymmetry, shows that the number of errors or correct answers do not involve top-down processing.

As for the SNT (Table 2), it is possible to note an improvement, especially in the LE, which shows that ATVD was effective at developing auditory closure skills, although it had not been directly stimulated. This finding is justified by the evidence that an improvement in attention, due to auditory training, facilitates the transfer of learning to tasks and skills not applied during training (20).

The ABR-click stimulus (21) is an auditory evoked potential of short-latency. It revealed a functional, structural modification of the auditory pathway at the brainstem level, which corroborates the behavioral change observed in temporal processing. It is hypothesized that ATVD reduces binaural processing asymmetry at the brainstem level, improving the quality of the acoustic signal of the frequency sent to the auditory cortex due to a structural change in the neural coding of the cochlea to the spiral ganglion (19).

In addition, the training conducted in this study simultaneously stimulated both ears with the same stimulus that is used a binaural condition. In this case, the contralateral pathway suppresses the ipsilateral pathway through the efferent corticofugal pathways (20). The suppression of the ipsilateral ascending pathways by the descending pathways prevents a bottleneck at the cortical level. Importantly, the brain and ear should be considered a single functional system with the ability to modify the representation of sounds in the cortex and modulate the information that comes out of the cochlea (22).

The origin of FFR includes multiple subcortical regions (23) and has seven characteristic peaks known as V, A, C, D, E, F, and O. The V, A, and C waves comprise its onset portion, which correspond to the plosive consonant /d/ and consonant-to-vowel transition /a/. The sustained portion FFR concerns the fundamental frequency (F_o_) and the formant transition of the consonant-vowel harmonic structure (24). The kind of stimulus used in this research caused a reorganization of the neural networks with respect to perception of the sustained portion of the stimulus since it spent more neural recruitment, contributing to the increase in the E wave latency.

The E wave latency in FFR showed a significant increase from M1 to M2 and a decrease in wave E from M2 to M3. However, M1 remained with a lower mean. Three hypotheses should be considered in relation to the above findings. First, there may have been a transient increase in E wave latency due to the increase in neuronal activity, and as there was only a short time between the end of the intervention and the reevaluation, this affected the results (25). Second, the initial values to the E wave latency were already close to normal, thus reducing the margin for better performance (26). Finally, latency measures and amplitude do not seem to be the most appropriate for assessing plasticity at a cortical level (25), as other studies have also found no significant changes in the FFR after auditory training (27,28).

Regarding LLAEP, the P3 component showed a significant increase in amplitude in the LE, a change that remained over time, as evidenced in the reassessment after 3 months of the intervention. It is known that amplitude is related to the magnitude of synaptic activity involved during perceptual processing of acoustic stimuli (12,29).

The results of this research show the effectiveness of binaural stimulation and temporal processing. Based on the findings reported above and to assist in planning the therapeutic approach to CAPD, it is suggested that binaural processing skills should be stimulated prior to training of DL to stimulate the brainstem to first establish the basis for more complex auditory processes involving the cortex (15).

### Clinical limitations

The assessment of the effectiveness of a binaural auditory training program with vocal duets was performed, and its effectiveness was evidenced after a maximum of 10 hours in 10 adult participants. Nevertheless, to validate the type of training used and investigate its clinical applicability, further studies should be carried out in other age groups and populations with a larger sample size.

## CONCLUSION

There was an improvement in the auditory abilities of temporal ordering, auditory figure-ground, and auditory closure with evidence from auditory evoked potentials and the results of behavioral tests, whose outcomes were maintained after 3 months.

## AUTHOR CONTRIBUTIONS

Picinini TA was responsible for the design of the study, data collection, database compiling, data analyses and interpretation, manuscript writing, review and final drafting. Sperança S was responsible for designing the study, collecting and analyzing the data, and revising the final manuscript drafting. Pereira LD advised on the study and was involved in the data analyses, manuscript writing, review and final drafting.

## Figures and Tables

**Table 1:** Mean and SE for the correct answers (ear-wise) in the DCVT in the NF, FR, and FL conditions and *p*-values for comparison at M1, M2, and M3.

	M1 (n=10)	M2 (n=10)	M3 (n=8)	*p* Least significant
Variables	Mean±SE	Mean±SE	Mean±SE	difference test
DCVT NF				
RE	13.4±1.0	12.2±0.9	12.5±0.7	0.499
LE	5.7±0.9^a^	6.5±0.8^ab^	7.9±0.7^b^	0.023*
Errors	4.9±0.7	5.2±0.6	3.6±0.7	0.083
Impaired	4 (40%)	4 (40%)	1 (12.5%)	0.266
FR				
RE	15.0±0.7	15.3±0.8	15.6±0.8	0.740
LE	4.5±0.8	4.2±0.7	4.0±0.6	0.882
Errors	4.6±0.5	4.5±0.7	4.4±0.7	0.795
Impaired	5 (50%)	4 (40%)	3 (37.5%)	0.718
FL				
RE	6.1±0.9	7.4±1.3	6.9±0.7	0.410
LE	11.3±0.9	12.2±1.1	11.8±0.5	0.744
Errors	6.6±0.8^b^	5.0±0.5^ab^	4.9±0.6^a^	0.007*
Impaired	6 (60%)	5 (50%)	6 (75%)	0.356

SE: standard error; RE: right ear; LE: left ear; DCVT: dichotic consonant-vowel test; NF: non-forced; FR: forced right; FL: forced left; ATVD: auditory training with vocal duets; *Significant values (*p*≤0.05); ^a,b^: results with identical letters do not differ in the least significant difference test at 5% significance.

**Table 2:** Mean and SE for correct answers (ear-wise) in the SNT, FPT, and RGDT, with *p*-values for comparison at M1, M2, and M3.

	M1 (n=10)	M2 (n=10)	M3 (n=8)	*p* Least significant
Variables	Mean±SE	Mean±SE	Mean±SE	difference test
SNT RE (%)	85.2±2.9^a^	93.2±1.4^b^	91.0±2.0^b^	0.002*
Impaired	1 (10%)	0 (0%)	0 (0%)	0.292
SNT LE (%)	85.2±2.2^a^	92.8±1.5^b^	91.5±1.3^b^	<0.001*
Impaired	1 (10%)	0 (0%)	0 (0%)	0.292
FPT - *Humming* (%)	59.7±5.3^a^	79.4±4.2^b^	80.7±4.9^b^	<0.001*
Impaired	9 (90%)^b^	4 (40%)^a^	3 (37.5%)^a^	0.038*
FPT - Naming (%)	63.7±6.6^a^	81.7±3.5^b^	81.7±6.7^b^	<0.001*
Impaired	7 (70%)^b^	4 (40%)^a^	2 (25%)^a^	0.023*
RGDT Mean m/s	6.4±1.1	5.1±0.7	5.5±0.9	0.261
Impaired	1 (10%)	0 (0%)	0 (%)	

SE: standard error; RE: right ear; LE: left ear; SNT: speech-in-noise test; FPT: frequency pattern test; RGDT: random gap detection test; M1: moment 1; M2: moment 2; M3: moment 3; *Significant values (*p*≤0.05); ^a,b^: results with identical letters do not differ in the least significant difference test at 5% significance.

**Table 3:** Mean and SE for correct answers (ear-wise) in the N2 and P3 components of the LLAEP test and *p*-values for comparing progress at M1, M2, and M3.

Variables	M1 (n=10)	M2 (n=10)	M3 (n=8)	*p* Least significant difference test
N2				
Latency (ms) RE	185.6±10.3	201.2±11.3	211.5±16.0	0.242
Impaired	1 (10%)	1 (10%)	3 (37.5%)	0.118
Latency (ms) LE	200.9±16.9	198.1±14.2	189.6±28.7	0.312
Impaired	2 (20%)	2 (20%)	3 (37.5%)	0.258
P3				
Latency (ms) OD	289.3±6.3	284.1±8.8	290.5±14.1	0.402
Impaired	0 (0%)	1 (10%)	1 (12.5%)	0.565
Latency (ms) LE	287.2±11.5	282.8±7.6	263.7±39.4	0.753
Impaired	2 (20%)	0 (0%)	2 (25%)	0.175
Amplitude - RE	10.9±1.6	8.1±1.1	11.1±2.1	0.064
Amplitude - LE	8.4±1.9^a^	7.8±1.5^a^	10.3±1.8^b^	0.035*

SE: standard error; RE: right ear; LE: left ear; LLAEP: long-latency auditory evoked potentials; ATVD: auditory training with vocal duets; *Significant values (*p*≤0.05); ^a,b^: results with identical letters do not differ in the least significant difference test at 5% significance.

**Table 4:** Descriptive absolute latency (ms) and inter-peak (ms) values in ABR at M1, M2, and M3.

	M1 (n=10)	M2 (n=10)	M3 (n=8)	*p* Least significant
Variables	Mean (ms)±SE	Mean (ms)±SE	Mean (ms)±SE	difference test
RE latency				
Wave I	1.65±0.03	1.66±0.03	1.71±0.06	0.387
Impaired	2 (20%)	2 (20%)	3 (37.5%)	0.125
Wave III	3.91±0.08	3.94±0.05	3.89±0.06	0.077
Impaired	2 (20%)	1 (10%)	1 (12.5%)	0.344
Wave V	5.68±0.07	5.76±0.06	5.56±0.08	0.364
Impaired	1 (10%)	2 (20%)	0 (0%)	0.287
I-III Inter-peak	2.26±0.06	2.28±0.05	2.19±0.04	0.111
Impaired	2 (20%)	1 (10%)	0 (0%)	0.287
III-V Inter-peak	1.78±0.04^a^	1.83±0.04^b^	1.67±0.09^a^	<0.001*
Impaired	0 (0%)	0 (0%)	0 (0%)	-
I-V Inter-peak	4.04±0.07^a^	4.11±0.05^b^	3.86±0.08^a^	<0.001*
Impaired	0 (0%)	0 (0%)	0 (0%)	-
LE Latency				
Wave I	1.63±0.03^a^	1.62±0.04^a^	1.71±0.05^b^	0.035*
Impaired	2 (20%)	2 (20%)	2 (25%)	0.941
Wave III	3.96±0.09	3.96±0.06	3.92±0.09	0.499
Impaired	3 (30%)	2 (20%)	2 (25%)	0.153
Wave V	5.70±0.07	5.69±0.06	5.63±0.06	0.279
Impaired	1 (10%)	1 (10%)	0 (0%)	0.292
I-III Inter-peak	2.34±0.07^b^	2.35±0.05^b^	2.21±0.05^a^	<0.001*
Impaired	4 (40%)^b^	3 (30%)^b^	0 (0%)^a^	<0.001*
III-V Inter-peak	1.74±0.04	1.73±0.03	1.73±0.08	0.926
Impaired	0 (0%)	0 (0%)	0 (0%)	-
I-V Inter-peak	4.08±0.05	4.07±0.06	3.93±0.07	0.119
Impaired	0 (0%)	0 (0%)	0 (0%)	-

SE: standard error; *: significant *p*-value for comparison between ears at 5% significance; RE: right ear; LE: left ear; ABR: auditory brainstem response; ATVD: auditory training with vocal duets; ^a,b^: results with identical letters do not differ in the least significant difference test at 5% significance.

**Table 5:** Descriptive latency (ms) values of the FFR components and *p*-values for comparison of progress at M1, M2, and M3.

	M1 (n=10)	M2 (n=10)	M3 (n=8)	*p* Least significant
Variables	Mean±SE	Mean±SE	Mean±SE	difference test
Latency				
Wave V	7.07±0.11	7.10±0.08	7.10±0.10	0.866
Impaired	3 (30%)	4 (40%)	2 (25%)	0.493
Wave A	8.17±0.23	8.30±0.14	8.66±0.31	0.334
Impaired	2 (20%)	4 (40%)	4 (50%)	0.124
D	23.5±0.3	23.8±0.7	23.3±0.2	0.695
Impaired	4 (50%)	6 (66.7%)	2 (25%)	0.092
E	32.2±0.3^a^	32.8±0.4^b^	32.6±0.3^ab^	0.002*
Impaired	4 (44.4%)	5 (55.6%)	4 (50%)	0.820
F	40.7±0.3	40.7±0.3	41.0±0.3	0.750
Impaired	5 (62.5%)	5 (50%)	5 (62.5%)	0.369
O	48.6±0.1	47.9±0.8	48.8±0.2	0.201
Impaired	0 (0%)	2 (20%)	1 (12.5%)	0.102
*Slope*	0.35±0.04	0.33±0.05	0.48±0.09	0.444
V-A Complex	0.36±0.05	0.38±0.05	0.35±0.06	0.657

SE: standard error; FFR: frequency following response; ATVD: auditory training with vocal duets, *Significant values (*p*≤0.05); a,b: results with identical letters do not differ in the least significant difference test at 5% significance.
